# Assessing Patient Preference for Specialty Training When Undergoing Cosmetic Injectables in a Resident Run Clinic

**DOI:** 10.1093/asjof/ojaf018.013

**Published:** 2025-05-13

**Authors:** Ashraf A Patel, Kaylee Scott, Sydney Somers, Cori A Agarwal, Renato Saltz, Courtney Crombie

**Affiliations:** University of Utah, Salt Lake City, UT; University of Utah, Salt Lake City, UT; University of Utah, Salt Lake City, UT; University of Utah, Salt Lake City, UT; University of Utah, Salt Lake City, UT; University of Utah, Salt Lake City, UT

## Abstract

**Goals/Purpose:**

Resident-run clinics (RRCs) are an integral component of aesthetic plastic surgery training with 60-70% of plastic surgery residency programs having a dedicated RRC. These clinics offer unique advantages to both patients and residents. For residents, RRCs help enhance procedural autonomy and training within aesthetic surgery with faculty supervision. For patients, cosmetic services are offered at a reduced rate with low complications and a high degree of patient satisfaction. At certain institutions, RRCs have been developed to increase resident exposure to nonsurgical methods of facial rejuvenation, through neuromodulator and soft tissue filler injections, either as a separate entity or engrained within a resident cosmetic surgery clinic. The cosmetic injection landscape is being increasingly widened as practitioners of a variety of training backgrounds (doctors, nurses, advanced practice providers (APPs), aestheticians) and specialties offer these services. Despite this changing landscape, injection experience remains an important part of plastic surgery training, governed by case minimum requirements for graduation. Although there is well-documented support from program directors and residents regarding the effectiveness of RRCs, there is need for further evaluation of these clinics from the patient's perspective, specifically with regard to injector characteristics. The purpose of this study is to assess patient preferences with regards to provider professional background, level in training, and specialty training among patients receiving injections at an RRC.

**Methods/Technique:**

This study surveyed patients between 2022-2024 who presented to University of Utah’s weekly RRC that offers cosmetic injectables. Patient characteristics such as whether patients had previously undergone cosmetic injections, a history of cosmetic surgery, and prior work experience in the healthcare field were collected. Patients were asked to rank their preference for provider level and type of specialty training. Specialties included Plastic Surgery, Otolaryngology/ENT/Facial Plastics, Ophthalmology/Oculoplastics, Dermatology, Internal Medicine, other residency training, and no residency training. Patients were also asked to rank their preference on the type of medical training their provider has, medical doctor (MD), osteopathic doctor (DO), Nurse Practitioner (NP), Physician Assistant (PA), Registered Nurse (RN), or Licensed Aesthetician. Additionally, patients were asked which resident post graduate year (PGY) they preferred to do their injections and the motivating factors for choosing to receive treatments at RRC.

**Results/Complications:**

A total of 40 patients completed this survey. Of those patients, 80% had previously undergone treatment with a neuromodulator or hyaluronic acid filler, and 35% of patients reported they had a prior history of cosmetic surgery. A majority of patients (62.5%) were aware that providers from specialties other than plastic surgery may offer treatment with injectables. Patients who were also healthcare workers (47.5%) were more likely than patients who had no healthcare background (52.5%) to know that providers other than plastic surgeons offer injectable treatments (89.4% vs. 38.0%). When patients were asked to rank what specialty training patients preferred their injector to have, 94.5% ranked Plastic Surgery as their first preference. The most common second preference ranking was Dermatology (48.6%), followed by Otolaryngology/ENT (48.6%), Ophthalmology/Oculoplastics (48.6%), Internal Medicine (67.5%), other specialty training (100%), and the lowest preference was providers with no specialty training (100%). Patients reported that they preferred their injector to be a MD (100%), followed by a DO (97.5%), PA (55%), NP (52.5%), RN (52.5%), and aesthetician (52.5%). If the provider was an APP, patients first preference of specialty training experience for the NP/PA was in plastic surgery (95%), second preference was dermatology (47.5%), third preference was Otolaryngology/ENT (42.5%), fourth preference Ophthalmology/Oculoplastics (47.5%), fifth preference Internal Medicine (75%), and sixth preference no specialty training (97.5%). With regards to postgraduate year (PGY) level of resident training, patients preferred the most senior residents (PGY-6 and PGY-5) to perform their injections, followed by PGY-4s or PGY 3s, and PGY-2s and PGY-1s. Cost was the primary reason patients sought treatments at the RRC.

**Conclusion:**

Overall, patients undergoing cosmetic injectables in our cohort had the strongest preference for providers to have a MD or DO degree and formal plastic surgery residency training. When the provider was an APP, they preferred that the APP had prior experience within the field of plastic surgery. At our RRC, all patients preferred having injectables done by residents of the highest postgraduate training year. Low cost was the primary motivator for patients choosing to undergo injections in our resident run clinic; however, some patients felt senior plastic surgery residents may have more frequent experience performing injections compared with plastic surgery attendings. We believe this data will be beneficial for establishing and expanding RRC offering injectables. Nearly half of our patients were not aware that injectables can be offered by providers who may not have formal plastic surgery training, highlighting a significant knowledge gap and area of improvement for patient education, safety, and advocacy.

